# The natural history of pedal puncture wounds in diabetics: a cross-sectional survey

**DOI:** 10.1186/1471-2482-11-27

**Published:** 2011-10-17

**Authors:** Jeffrey M East, Curtis B Yeates, Hector P Robinson

**Affiliations:** 1 Department of Surgery, Cornwall Regional Hospital (CRH), Montego Bay, Jamaica; 2 Department of Surgery, Radiology, Anesthesia and Intensive Care, University of the West Indies, Mona, Kingston 6, Jamaica; 3 Department of Medicine, CRH, Montego Bay, Jamaica; 4 Department of Medicine, University of the West Indies, Mona, Kingston 6, Jamaica; 5 41 Church Street, Montego Bay, Jamaica

## Abstract

**Background:**

Surgeons usually witness only the limb-threatening stages of infected, closed pedal puncture wounds in diabetics. Given that this catastrophic outcome often represents failure of conservative management of pre-infected wounds, some suggest consideration of invasive intervention (coring or laying-open) for pre-infected wounds in hope of preventing contamination from evolving into infection, there being no evidence based guidelines. However, an invasive pre-emptive approach is only justifiable if the probability of progression to catastrophic infection is very high. Literature search revealed no prior studies on the natural history of closed pedal puncture wounds in diabetics.

**Methods:**

A survey was conducted via an interviewer-administered questionnaire on 198 adult diabetics resident in the parish of St. James, Jamaica. The sample was selected using a purposive technique designed to mirror the social gradient and residential distribution of the target population and is twice the number needed to detect a prevalence of puncture wounds of 14% with a range of 7-21% in a random sample of the estimated adult diabetic population.

**Results:**

The prevalence of a history of at least one closed pedal puncture wound since diagnosis of diabetes was 25.8% (CI; 19.6-31.9%). The only modifiable variable associated at the 5% level of significance with risk of pedal puncture wound, after adjustment by multivariable logistic regression, was site of interview/paying status, a variable substantially reflective of income more so than quality-of-care.

Of 77 reported episodes of closed pedal puncture wound among 51 participants, 45.4% healed without medical intervention, 27.3% healed after non-surgical treatment by a doctor and 27.3% required surgical intervention ranging from debridement to below-knee amputation. Anesthetic foot (failure to feel the puncture) and sole of the forefoot as site of puncture were the variables significantly associated with risk of requiring surgical intervention.

**Conclusions:**

That 72.7% of wounds healed either spontaneously or after non-surgical treatment means that routine, non-selective surgical intervention for pre-infected closed pedal puncture wounds in diabetics is not justifiable. However the subset of patients with an anesthetic foot and a wound on the sole of the forefoot should be marked for intensive surveillance and early surgical intervention if infection occurs.

**Trial Registration:**

ClinicalTrials.gov: NCT01151891

## Background

Surgeons usually witness only the limb-threatening stages of infected puncture wounds of the feet in diabetics as surgical consultation at the earlier, pre-infection phase is uncommon. Given that this catastrophic outcome often represents failure of conservative management of pre-infected wounds, usually consisting of watchful waiting and antibiotics, some wonder if invasive intervention (coring or laying-open) for pre-infected wounds might not prove to be more effective in preventing contamination from evolving into infection. After all, debridement or amputation just ahead of advancing sepsis almost always controls invasive infection in a diabetic foot and therefore debridement of contaminated tissue ought to prevent evolution into infection. A careful literature search revealed no evidence-based recommendations among international consensus guidelines for initial management of fresh, uninfected, closed puncture wounds of the feet in diabetics [1-3]. Indeed international guidelines seem to have largely ignored pedal puncture wounds as a significant cause of serious lower limb infection in diabetics, concentrating almost exclusively on the role of neuropathic ulceration. The reason for this is unclear but it could be that puncture wounds of the feet may be a proportionally less significant precipitant for serious lower limb infections among diabetics in developed countries than they appear to be in developing countries.

Pre-emptive debridement (coring or laying open) of closed pedal puncture wounds has been tried in non-diabetics but has an unfavourable risk-benefit profile in this group because of a very high rate of spontaneous healing, with or without antibiotics [4,5]. However, this unfavourable risk-benefit profile may not be applicable to diabetics, who manifest reduced capacity to prevent contamination evolving into infection. Certainly, infected puncture wounds are more likely to follow a catastrophic course in diabetics than in non-diabetics [6].

Pre-emptive debridement and any randomized trial to test this approach in the management of fresh, uninfected, closed puncture wounds of the feet in diabetics is not ethically justifiable unless it is known that the probability of progression to severe infection is very high if untreated or treated by non-invasive measures alone, because invasive treatment carries some risk of worsening the problem by providing a portal for additional contamination and increasing discomfort in patients whose wound might not have become infected in the first place [5]. One study on the natural history of pedal puncture wounds in the general population found that such wounds progressed to infection in only 6.4% of cases [5]. Surprisingly, no similar studies, on the natural history of such wounds among diabetic patients, were identified in the literature. Lavery et al [7], in a closely followed cohort of 1,666 diabetic patients, demonstrated that of 50 pedal wounds due to trauma, 36 became infected and 14 did not but puncture wounds were not disaggregated within the trauma category and no details were given regarding initial treatment of uninfected wounds. The question as to whether there might be pedal puncture wounds which heal without medical intervention was not addressed.

The Cornwall Regional Hospital (CRH) in Montego Bay, St. James, Jamaica, is the largest of three public hospitals and the only tertiary level referral facility serving a population of 302,927 people over 15 years old within the catchment area of the Western Regional Health Authority (parishes of St. James, Hanover, Westmoreland and Trelawny), 116,839 of whom live in St. James [8]. At this hospital there have been an average of 41 lower limb amputations in diabetics in each of the five years since 2005, and this does not include minor amputations and debridement performed in ward treatment rooms. Although the distribution of proximate precipitating factors for infection has not been prospectively studied in this specific population, there is an experiential impression that a substantial number of pedal infections are initiated by closed penetrating wounds. Wright-Pascoe et al [9], in a retrospective record review at the University Hospital of the West Indies, Kingston, Jamaica, observed a prevalence of puncture wounds of 11.8% among 44 patients with the "diabetic foot" but it is unclear whether the study population had active infections or just contaminated ulcers.

The primary aim of this study is to determine the natural history of closed pedal puncture wounds among adult diabetics residing in the parish of St. James, Jamaica, by interviewing a large sample across all social classes. Specific, primary objectives are to determine the prevalence of closed pedal puncture wounds in this population as well as the outcome of such wounds. Secondary objectives include identification of variables associated with the risk of sustaining a closed pedal puncture wound, which may assist in formulation of risk reduction strategies, and identification of risk factors predictive of infection and unfavourable outcome, which may be helpful in formulation of guidelines for the management of pre-infected wounds.

## Methods

The research protocol was approved by the Ethics and Medico-Legal Affairs Advisory Panel of the Ministry of Health in Jamaica and the Ethics Committee of the Faculty of Medical Sciences/University Hospital of the West Indies, University of the West Indies at Mona, Kingston, Jamaica. Informed consent was received from all participants.

The study is a non-randomized cross-sectional survey. Data were extracted from an interviewer-administered questionnaire (available on request) which also recorded data relevant to a separate nested study on the effect of diabetes education and self-reported compliance with diabetes treatment on the prevalence of lower limb infections. The questionnaire was created de novo and was pre-tested twice on a group of local surgical consultants and residents used to communicating daily with patients in all social strata in public and private hospitals and therefore aware of questions likely to be misinterpreted within each stratum. The final instrument was considered by the group to have face and content validity sufficient to minimize interpretive bias. Though desirable, it was not logistically practical to pre-test the questionnaire among the different social classes within the target population. The interviewer, a Jamaican undergraduate non-medical university student, was then trained by the principal investigator and was instructed to use dialect tactfully to explain any questions which the subjects did not appear to understand.

Subjects eligible for interview were patients of either gender eighteen years and older with established diabetes mellitus and resident within the parish of St. James, Jamaica. Exclusions were patients with altered mental status (eg, dementia, delirium) or who were otherwise too ill to tolerate the interview. Given that the prevalence of diabetes mellitus among the adult population of Jamaica is estimated at 13% [10], there ought to be about 15,190 diabetics among the 116,839 inhabitants of St. James who are over 15 years old. This figure is used in the sample size calculation because the population of St. James over 18 years old is not disaggregated in the demographic statistics publication [8], with the assurance that the calculated sample size would not be less than required to detect the chosen parameter in the lesser over-eighteen population.

Exhaustive literature search revealed no prior epidemiological research into the incidence or prevalence of closed pedal puncture wounds among diabetics in general in any population to serve as a guide for calculation of sample size. An estimate of 14% was arbitrarily adopted, partially based on an assumption that the prevalence of a history of closed pedal puncture wounds among the target population was likely to be higher than the 11.8% prevalence reported by Wright-Pascoe et al [9] among patients with the diabetic foot at a Jamaican hospital. The number of randomly selected subjects that need to be interviewed from an estimated diabetic population of 15,190 to detect a prevalence of pedal puncture wounds of 14% at the 95% confidence level, allowing for a range of 7 - 21%, is 94 (Epi-Info Version 3.4.1). Since it was not possible to select a random sample, the sample size was doubled to 188 to reduce the inevitable effect of selection bias.

Subjects were selected via a purposive sampling technique, meant to mirror the social class spectrum and urban-rural distribution of the residents of St. James. The number of patients targeted for interview was greater than 188 to allow for refusals and post-interview exclusions. Eighty three interviewees were selected from the outpatient clinics of the internal medicine, general surgery and ophthalmology services at the CRH, 21 from the main public health center (health centers are primary care facilities) in Montego Bay, capital city of St. James, 33 from 3 suburban health centers, 22 from 2 deep rural health centers and 42 from the private practices of 2 of the participating researchers. Interviewees were subsequently classified into urban/suburban versus rural on the basis of home address, regardless of the site at which they were interviewed. The interviewer was asked to exercise deliberate selection bias in favour of male subjects (by consenting all eligible male patients at an interview site before approaching females) in the hope of minimizing the effect of the known reluctance of men in Jamaica to seek health care [11], granted, nevertheless, that the prevalence of diabetes is higher among females [12].

Variables extracted from the questionnaire were age at last birthday, gender, those which allowed classification by social class according to the United Kingdom Registrar General's Social Classification [13], site interviewed (public versus private facility), current occupation, current pastime, current glycemic control (defined for the purposes of this study as a "mild lack of control", according to Guidelines for the Management of Diabetes, Ministry of Health [14], in the 3 months preceding the interview), time since being diagnosed with diabetes, clinically significant current comorbidity, self-reported current compliance with oral medication, insulin dependence, self-reported compliance with insulin therapy, exposure to pedal injury avoidance education, pedal injury avoidance behaviour, exposure to education in respect of importance of visiting a health facility within 24 hours after pedal puncture and occurrence of closed pedal puncture wound since being diagnosed with diabetes. Subjects who had sustained more than 4 closed puncture wounds were asked to recall only the 4 with the worst outcomes as it was felt that accurate recall beyond this number would be unlikely. Additional variables were recorded for each episode of a closed pedal puncture wound, namely implement causing puncture, location of the implement at the time of puncture, self-assessed depth of wound, foot affected, part of foot affected, activity at the instant of puncture, footwear at the instant of puncture, whether puncture was felt at the instant it happened (a proxy test for sensory neuropathy), home remedies applied, compliance with diabetes treatment at time of puncture, smoking status at time of puncture, health seeking behaviour after puncture, treatment at first encounter with a doctor and outcome of puncture wound. Unfortunately, some variables having plausible association with the risk of poor outcome after pedal puncture wounds, such as insulin dependence, glycemic control and arterial insufficiency, were not measurable in relation to each episode of puncture in this survey of self-reported experience. Extracted data were entered into a STATA 11 database for analysis.

Two versions of the database were created, one with wide coding for analysis of subject-based data and the other with long coding for analysis of event-based (puncture wound) data. The main statistical output is the frequency distribution of the prevalence of a history of closed pedal puncture wound stratified by outcomes and plausibly associated variables. The frequency distribution of episodes of closed pedal puncture wound by outcomes and plausibly associated variables is also tabled. Stepwise logistic regression is used to determine whether any of the risk factors measured are associated either with the risk of having sustained a closed pedal puncture wound or with the risk of poor outcome.

## Results

Of the 208 patients approached for interview, only 9 (4.3%) refused to participate, thereby ensuring minimal non-response bias. One hundred and ninety nine interviews were therefore completed. One participant was subsequently excluded because of historical evidence of dementia, leaving 198 interviews for analysis.

The records of 65 of the interviewees regularly treated at CRH were examined with a view to determining the concurrent validity of the questionnaire in respect of verifiable items. Specifically, the phi correlation coefficient for interviewee-reported occurrence (or not) of closed puncture wounds versus historical accounts in the hospital records is 0.93 (P < 0.001). Of the 8 patients among the 65 retrieved records who correctly reported that they were hospitalised as a direct consequence of a puncture wound, only one reported the outcome incorrectly (phi = 0.65, P = 0.064).

Fifty one participants (25.8%; CI: 19.6-31.9%) reported having sustained at least one closed pedal puncture wound since being diagnosed with diabetes mellitus, with 15 reporting 2 or more (range 2-10). Table 1. illustrates the distribution of relevant variables by history of closed pedal puncture wound.

**Table 1:** Distribution of relevant variables by history of closed pedal puncture wound among 198 subjects.

	History of closed pedal puncture wound		
	No	Yes	**P-value (chi**^**2 **^**or t-test)**
**Number of cases **(%)	147(74.2%)	51(25.8%)	
95% CI	68.1-80.4%	19.6-31.9%	
**Age **- mean(range)	63.4(29-97)	59.3(32-81)	0.028
**Gender **- no.(%) female	104(70.8%)	34(66.7%)	0.589
**Social class **- I,II&III(%)	39(26.5%)	7(13.7%)	0.062
**Paying status **- private (%)	39(26.5%)	4(7.8%)	< 0.01
**Residence **- no.(%) rural	54(36.7%)	26(51%)	0.074
**Current occupation **- sedentary (%)	105(71.4%)	35(68.6%)	0.71
**Current recreation **- sedentary (%)	105(71.4%)	38(74.5%)	0.67
*Current glycemic control - no.(%)	75/120(62.5%)**	18/43(41.9%)**	0.019
**Duration diabetes **- > 10 yrs(%)	85(57.8%)	40(78.4%)	< 0.01
**Significant current comorbidity - **no.(%)	113(76.9%)	36(70.6%)	0.37
**Currently compliant with Rxed oral meds - **no.(%)	130/140(92.9%)	42/49(85.7%)	0.13
**Insulin dependence - **no.(%)	29(19.7%)	20(39.2%)	< 0.01
**Currently compliant with Rxed insulin - **no.(%)	29/29(100%)	19/20(95%)	0.22
***Foot protection education - no.(%)yes	122(83%)	46(90.2%)	0.22
**Specific foot protection education - **no.(%)yes	115/122(94.3%)	44/46(95.7%)	0.72
**Currently compliant with foot protection - **no.(%)	145(98.6%)	50(98%)	0.76
**Early post-puncture treatment education - **no.(%)	93(63.3%)	36(70.6%)	0.34

*Glycemic control defined here as "mild lack of control" (fasting blood glucose≤8.9 mmol/L, random blood glucose≤11.1 mmol/L, 2 hr postprandial glucose≤10 mmol/l or HbA1c≤7.5%) according to the Guidelines for the Management of Diabetes, Ministry of Health (10), in the 3 months prior to interview.

**27 values (18.4%) for "current glycemic control" were missing among the group without puncture and 8 (15.7%) among the group with puncture (P = 0.67)

***Foot protection education was more comprehensively disseminated among public (87.1%) than private patients (76.7%), though not statistically significant (P = 0.094).

The variable with the strongest association with risk of puncture wound is site of interview (that is, private or public patient status). This was therefore used as the main predictor variable in a logistic regression model of effect on risk of having sustained a puncture wound. Only variables associated with risk of puncture wound at a P-value of less than 0.1 were considered likely confounders to be included in the regression model. Social class was dropped, despite a P-value of 0.062 for association with risk of puncture wound, because of high correlation with site of interview (P < 0.001) plus the fact that it is a less objectively measured variable than site of interview. Insulin dependence, residence (urban/suburban versus rural) and current glycemic control (following multiple imputation of missing values) were all dropped from the final logistic regression model after stepwise, forward inclusion because of P-values exceeding 0.1. Table 2. displays the final logistic regression model for the effect of site of interview/paying status (private) on risk of having sustained a puncture wound. Site of interview/paying status, age and duration of diabetes remain significant effectors in this model. Despite the high degree of correlation between social class and site of interview/paying status, the effect of social class on risk of sustaining a puncture wound remained insignificant at the 5% level (P = 0.089) if it replaces site of interview/paying status in the final regression model.

**Table 2:** Multivariable logistic regression model for effect of paying status (private) on risk of puncture wound.

Variable	Odds Ratio	95% CI for OR	P-value
Paying status (private)	0.256	0.084 to 0.775	0.016
Age	0.967	0.938 to 0.997	0.032
Duration diabetes (> 10 yrs)	3.207	1.469 to 6.999	0.003

"Social class" was dropped from the model, despite a P-value of 0.062 for association with risk of puncture wound, because of high correlation with site of interview (P < 0.001). "Social class" remains an insignificant effector at the 5% level (P = 0.089) if it replaces site of interview/paying status in the final regression model.

Among the 51 participants reporting at least 1 puncture wound, worst outcome of any puncture wound in 33 (64.7%) patients was healing without surgical intervention and in the remaining 18 (35.3%) was infection requiring debridement and/or amputation. All of the variables in Table 1. were tested against worst outcome and only 3 were found to be associated at a P-value of less than 0.1, namely significant current comorbidity, insulin dependence and early post-puncture treatment education. In a logistic regression model including all 3 variables only early post-puncture treatment education maintained a significant (positive) effect on poor "worst outcome". This ostensibly spurious effect suggests that early post-puncture treatment education was likely acquired more commonly after rather than before the puncture wound had occurred. Failure of statistical association notwithstanding, it is noteworthy that of only 4 private patients reporting having sustained a puncture wound, none were among the group requiring debridement and/or amputation

Given that data were collected on a maximum of 4 episodes of puncture wound per participant reporting its occurrence, there are 77 episodes for analysis. Of these, 35(45.4%) healed without involvement of any medical professional, 21(27.3%) healed after non-surgical treatment by a doctor and 21(27.3%) required debridement and/or amputation. Among the group of 21 episodes requiring debridement and/or amputation, 11(52.4%) healed after limited debridement alone, 5(23.8%) after amputation of unspecified digits, 2(9.5%) after below knee amputation and 3(14.3%) remained unhealed after debridement alone at the time of the interview. These sub-groups are individually too small to permit any meaningful risk analysis in relation to level of amputation, hence their inclusion into a single group requiring surgical treatment in the analysis which follows. Table 3. illustrates the distribution of relevant variables by outcome of episode of closed pedal puncture wound with unordered categorical variables dichotomized by most frequent category or category with the most significant association with outcome. A version of Table 3. displaying all unedited categories of the relevant variables is accessible as additional file 1.

**Table 3:** Distribution of relevant variables by outcome of 77 episodes of closed pedal puncture wound.

	Outcome of closed pedal puncture wound			
	Healed, no doctor	Healed, non-surgical Rx	Debridement, amputation	P-value (chi^2 ^or t-test)
**Number of cases **(%)	35(45.4%)	21(27.3%)	21(27.3%)	
95% CI	34.1-56.8%	17.1-37.4%	17.1-37.4%	
*Infected - no.(%)	0	5(23.8%)	21(100%)	< 0.001
Implement of puncture** - no.(%): nail/metal fragment	10(28.6%)	10(47.6%)	12(57.1%)	0.089
**Location implement **- no.(%): on the ground	31(88.6%)	17(80.6%)	18(85.7%)	0.73
**Depth of wound - **no.(%): deep	7(20%)	6(28.6%)	9(42.9%)	0.186
**Limb - **no.(%): right***	12/18(66.7%)	10/18(55.6%)	12/21(57.1%)	0.76
**Part of foot affected - **no.(%): anterior sole	12(34.3%)	9(42.9%)	16(76.2%)	0.008
**Activity at time of puncture**! - no.(%): nothing special	13(37.1%)	15(71.4%)	10(47.6%)	0.045
**Footwear at time of puncture - **no.(%): slippers only	26(74.3%)	16(76.2%)	17(81%)	0.848
**Puncture not felt - **no.(%)	2(5.7%)	5(23.8%)	10(47.6%)	0.001
**Home remedy? **- no.(%): yes	33(94.3%)	13(61.9%)	13(61.9%)	0.004
**Type home remedy**!! - no.(%): disinfectant	12/33(36.4%)	7/13(53.9%)	3/13(23.1%)	0.265
**Unprescribed oral antibiotic? **- no.(%): yes	0	1(4.8%)	0	0.236
**Compliant with diabetes Rx at time of puncture - **no.(%): yes	34(97.1%)	21(100%)	18(85.7%)	0.08
**Smoking status at time of puncture - **no.(%): current	0	0	1(4.8%)	0.259
**Time from puncture to doctor's visit - **days (%): less than 3		19(90.5)	12(57.1%)	0.014

In 3 of the 5 episodes of infection which resolved without surgical intervention, the participant did not feel the puncture; in 2, the implement was a nail; in 4, the wound was thought to be deep and in 2, the part of the foot affected was the fore-sole. There were therefore no variables which allowed prediction of resolution of infection without surgical intervention.

*Infection defined as an issue of pus and/or appearance of redness and/or increased local temperature

**Other implements of puncture include thorn (11), glass fragment (9), needle (1), thumbtack (3), tip of a machete (3), stone fragment (3), wood fragment (2) and barbed wire (1). All punctures due to thorns healed without medical intervention.

***For 20 episodes, participants could not remember which foot was affected.

!Other activities at the time of puncture include job (18) and housework/gardening (21). No participant reported being involved in recreation at the time of puncture.

!!Other home remedies include "black dressing" (a tar based ointment) (14), antibiotic cream (2), antibiotic powder (4), black shoe polish and kerosene.

The outcome variable was dichotomized, to facilitate logistic regression, into episodes of puncture wound requiring surgical treatment versus those healing without surgical intervention. All variables from Table 3. associated with outcome of episode of puncture wound at a P-value of < 0.1 were candidates for inclusion in a logistic regression model of effect on outcome. "Infected" was dropped from the model by STATA because it predicted failure perfectly (all episodes requiring surgery were infected) and compliance with diabetes treatment was dropped because it predicted success perfectly (in 98.2% of episodes which healed without surgery, patients claimed that they were compliant at the time the puncture occurred, versus 85.7% for episodes requiring surgery). Implement of puncture (nail or metal fragment), activity at time of puncture wound and whether home remedy was applied or not were dropped after stepwise inclusion in the model (with whether or not the puncture was felt as the main predictor variable) because of P-values greater than 0.1. Table 4. displays the final logistic regression model for the effect of not having felt the puncture wound on risk of poor outcome (that is, need for surgical intervention). Puncture not felt, sole of forefoot as site of puncture and reporting to a doctor more than 3 days after puncture are the only significant effectors in this model.

**Table 4:** Multivariable logistic regression model for effect of not having felt puncture on risk of poor outcome.

Variables	Odds Ratio	95% CI for OR	P-value
Puncture not felt	6.64	1.2 to 36.7	0.03
Front sole punctured	5.97	1.16 to 30.69	0.033
Visited Dr. > 3 days after event	10.34	1.47 to 72.9	0.019

"Infected" was dropped from the model because this variable predicted failure perfectly (all episodes requiring surgical intervention were judged to be infected by patients).

"Compliance with diabetes treatment" was dropped because it predicted success perfectly (in 98.2% of episodes which healed without surgery, patients claimed that they were compliant at the time the puncture occurred, versus 85.7% for episodes requiring surgery.

## Discussion

The study reveals a prevalence of 25.8% (CI: 19.6-31.9%) for history of ever having sustained a significant pedal puncture wound since receiving a diagnosis of diabetes mellitus among a representative sample of adult diabetics living in the parish of St. James, Jamaica. The only modifiable variable associated with risk of having sustained a pedal puncture wound after adjustment in a multiple logistic regression model (Table 2) is site of interview/paying status. Site of interview/paying status is ostensibly a measure of the quality of private versus public care and of income (patients do not access severely overcrowded public facilities if they can afford private care). It is doubtful that there is any significant difference in quality between private and public care in relation to foot care or injury prevention instruction. Neither type of facility offered podiatry services and foot care and injury prevention education were better distributed among public patients (though not to a statistically significant extent). In this study, site of interview/paying status is therefore more substantially a proxy measure of income and, by extension, living conditions, than it is of quality of care. It was not considered prudent to ask specific questions about income during the interview because of known respondent resistance to such questions [15].

Knowledge of the necessity for diabetics to take specific measures to protect their feet was not statistically associated with the risk of having sustained a puncture wound nor was early post-puncture treatment education associated with risk of poor outcome. Effective delivery of foot protection education has the potential to significantly reduce the incidence of puncture wounds and improve outcome and this tool is of particular importance in resource-challenged countries where increased personal income and specialist foot care services are not attainable national goals in the short term. It is impossible to determine the possible preventive effect of education from a survey of this type since the knowledge may have been acquired after the fact (of the puncture). In our cohort, specific education regarding the need to protect the feet is high among public as well as private patients but penetration of early post-puncture treatment education demands considerable improvement. With concerted effort to provide this information to all newly diagnosed diabetics at all treatment sites, penetration of education should approach 100%.

How effective education is in reducing puncture wound incidence and improving outcome depends on how it is delivered. Malone et al [16] demonstrated that specific foot protection education can substantially reduce the risk of amputation in diabetics. On the other hand, Lavery et al [7] expressed surprise that the risk of pedal infection resulting from trauma remained high in their cohort of diabetics despite intensive education. Price, in alluding to the likely effectiveness of diabetes education in preventing foot infections, has suggested that education on its own will not necessarily lead to behaviour change [17]. Certainly, foot protection education needs to be specific and must include attention to person behaviour as well as the environment - 85.7% of pedal puncture episodes in this study were caused by implements lying around in the yard (Table 3.).

That 45.4% (CI: 34.1-56.8%) of 77 reported episodes of closed pedal puncture wounds healed without interaction with the formal medical establishment is new information. These injuries which heal without medical intervention should not be dismissed as likely to have been trivial. Although patients are notoriously inaccurate in their assessment of puncture wound depth [4], it is unlikely that even wounds self-assessed as superficial were trivial since an unsubstantial wound is unlikely to have been recalled by participants, especially if it did not become infected. In addition, 57.1% of the 21 episodes of puncture wound which eventually required surgical intervention (including major amputation) were self-assessed as being superficial and superficial trauma is known to be capable of causing severe lower limb infection in diabetics [18].

A total of 72.7% (CI: 62.5-82.9%) of wounds healed without requiring surgical intervention of any kind. That such a high proportion of wounds healed either spontaneously or after non-surgical treatment means that routine, non-selective surgical intervention for pre-infected closed pedal puncture wounds in diabetics is not justifiable. However, once infection had set in, the risk that the wound required surgical intervention was 80.8% (CI: 64.5-97%). There being no variables in this study which enable prediction of successful resolution of infection with antibiotics alone (that is, without need for surgical intervention) and given the known predilection of such infections in diabetics to smoulder beneath the surface and the potentially devastating consequences of treatment failure, it would seem to be prudent to consider, at a minimum, de-roofing of all infected pedal puncture wounds at the time infection is diagnosed.

Anaesthetic foot (failure to feel the puncture wound) and puncture wound to the sole of the forefoot, two of three variables associated with (and predictive of) poor outcome in this study (Table 4) should be assessed in every diabetic patient who presents with a pre-infected closed pedal puncture wound. Other variables known to be associated with the predisposition of diabetics to pedal infection after trauma, such as severity of peripheral arterial disease, glycemic control at the time of the injury, insulin dependence and characteristics of the wound itself, such as depth, none of which were objectively measurable in this study in relation to each episode of puncture, should also be assessed. Were they measurable, inclusion of these potential confounding variables in the final regression equation might have affected the values of the observed odds ratios for effect of anesthetic foot and site of puncture, but not enough to render the odds ratios insignificant, given that none can plausibly substantially explain the effect of either of these two variables. The significant association between poor outcome and presentation to a doctor after 3 days should not be misinterpreted as meaning that all patients who present beyond 3 days after the puncture wound are likely to have a poor outcome. What it is more likely to mean in the context of this study is that patients did not seek medical attention until after the wound became infected.

That an anesthetic foot is a risk factor for infection after pedal puncture in diabetics is established [19], this because of prolonged exposure to the offending implement due to the absence of a withdrawal response. Association between puncture wounds of the sole of the forefoot and subsequent serious infection has been identified in the general population [20], but this is the first report of the association being confirmed specifically among diabetics. Despite the high predictive capacity for poor outcome of a concurrence of failure to feel the puncture (anesthetic foot) and sole of forefoot as site of puncture identified in this study, it remains injudicious to recommend pre-emptive debridement of a pre-infected closed pedal puncture wound in these patients, in the absence of a prospective risk-benefit evaluation of this approach. However, these variables do mark such patients as candidates for heightened surveillance and for at least daily inspection for the appearance of clinical stigmata of infection, in addition to prophylactic antibiotics. Prophylactic antibiotic for pedal puncture wounds is of undetermined effectiveness in the general population [4] but until proven ineffective in diabetics, it would be foolhardy not to administer it. Debridement should be recommended to the patient on emergence of any clinical signs of infection, whether or not he/she is already receiving appropriate antibiotics. The reliability of heightened surveillance would be enhanced if a convenient, objective method for detecting early soft tissue infection were to be identified, rather than the often delayed onset of clinical and laboratory stigmata [21]. Ultrasonography is promising in this regard [22].

Despite employing standard procedures in this study to minimize the effect of the many potential sources of bias known to be inherent in the survey design, the design itself as well as failure to select a random sample would have affected the accuracy of the estimate of the prevalence of closed pedal puncture wounds in the target population. Nevertheless, a reasonable effort was made to sample all levels of the social gradient in crude proportion to their estimated distribution in the population. Moreover, the aim of the study was not so much to accurately determine the prevalence of puncture wounds as to explore the natural history of these injuries after they occur and in that the study was successful. That pedal puncture wounds in diabetics may have healed without medical intervention can only be determined by asking the patients, that is, by conducting a survey. Prospective determination would require a captive and cooperative cohort enrolled at the time of diagnosis of diabetes with detailed follow-up for many years.

Another limitation of the study was the fact that the questionnaire had to be interpreted by the interviewer in a non-standardized way to a significant proportion of the participants to ensure comprehension of the questions across social classes and varying levels of literacy, thereby minimizing interpretive bias. The impact of this on the overall validity of the questionnaire is impossible to assess but it is reassuring that at least in relation to the question about a history of pedal puncture wounds, the questionnaire achieved acceptable validity. Surprisingly, this problem has not been adequately addressed in the literature, although it must obviously affect the validity of surveys in multi-ethnic (different languages) and developing countries (highly variable levels of literacy and education). Although not addressing this specific concern, Subramanian et al [23] assessed the validity of self-reported morbidities in India and make a plea for a less dismissive view of health data obtained through self-reports from developing countries.

## Conclusions

This study unearths a relatively high prevalence of a history of closed pedal puncture wound of 25.8% (CI, 19.6-31.9%) among a representative sample of adult diabetics resident in the parish of St. James, Jamaica. The only modifiable variable associated with the risk of having sustained a pedal puncture wound, after adjustment in a multiple logistic regression model, was site of interview/paying status, a variable predominantly reflective of income rather than quality of care differences between private and public facilities. The preventive value of foot protection and early post-puncture treatment education was not assessable in this study but penetration of this critical information among the diabetic population, though reasonably high, needs to be improved.

That 45.4% of 77 reported episodes healed without involvement of the medical establishment and a further 27.3% healed after non-surgical treatment by a doctor means that a policy of routine, non-selective pre-emptive surgical intervention (coring or laying open) for pre-infected closed pedal puncture wounds in diabetics is not justifiable. Anesthetic foot (puncture wound not felt) and sole of the forefoot as site of puncture were strongly associated with risk of infection requiring surgical intervention at the 5% level of significance in a multivariable logistic regression model. These variables, in addition to other established risk factors which were not measureable in this study, such as peripheral arterial insufficiency, glycemic control and local wound characteristics, should mark diabetics with pre-infected pedal puncture wounds for at least a higher level of surveillance for progression to infection and early surgical treatment if it becomes necessary.

## Competing interests

The authors declare that they have no competing interests. Stipend and travelling allowance for the interviewer as well as stationery and computing resources were funded entirely by JME from personal resources after attempts to secure funding from one international and one local funding agency failed. The interviewer is the daughter of JME.

## Authors' contributions

All authors facilitated the acquisition of data and participated in the revision of the manuscript. JME conceived of the study, determined the design, trained the interviewer, performed data entry and statistical analysis, interpreted the data and drafted the manuscript. All authors read and gave final approval of the version submitted for publication.

## Pre-publication history

The pre-publication history for this paper can be accessed here:

http://www.biomedcentral.com/1471-2482/11/27/prepub

## Supplementary Material

Additional file 1 **Distribution of relevant variables by outcome of 77 episodes of closed pedal puncture wound, expanded version**. Version of Table 3. displaying all unedited categories of relevant variables. Click here for file
