# Supplementary material for: The natural history of pedal puncture wounds in diabetics: a cross-sectional survey
Source: BMC Surg. 2011 Oct 17;11:27. doi: 10.1186/1471-2482-11-27 (PMC3209435; doi:10.1186/1471-2482-11-27)
Supplement: Additional file 1 — Distribution of relevant variables by outcome of 77 episodes of closed pedal puncture wound, expanded version. Version of Table 3. displaying all unedited categories of relevant variables. [file 1471-2482-11-27-S1.DOC]

Table 3. Distribution of relevant variables by outcome of 77 episodes of closed pedal puncture wound, expanded version.

|  | Outcome of closed pedal puncture wound | | |  |
| --- | --- | --- | --- | --- |
|  | Healed, no doctor | Healed, non-surgical Rx | Debridement, amputation | P-value  (chi^2^ or t-test) |
| **Number of cases** (%) | 35(45.4%) | 21(27.3%) | 21(27.3%) |  |
| **Infected** - no.(%) | 0 | 5(23.8%) | 21(100%) | <0.001 |
| **Implement of puncture** – no.(%) |  |  |  |  |
| **Nail/Metal fragment** | 10(28.6%) | 10(47.6%) | 12(57.1%) | 0.007 |
| **Thorn** | 11(31.4%) | 0 | 0 |  |
| **Glass fragment** | 4(11.4%) | 3(14.3%) | 2(9.5%) |  |
| **Other*** | 4(11.4%) | 7(33.3%) | 4(19%) |  |
| **Don’t know** | 6(17.1%) | 1(4.8%) | 3(14.3%) |  |
| **Location implement** – no.(%) |  |  |  |  |
| **On the ground of yard** | 31(88.6%) | 17(80.6%) | 18(85.7%) | 0.63 |
| **On the floor** | 2(5.7%) | 1(4.8%) | 0 |  |
| **Other** | 2(5.7%) | 3(14.3%) | 3(14.3%) |  |
| **Depth of wound –** no.(%): deep | 7(20%) | 6(28.6%) | 9(42.9%) | 0.186 |
| **Limb –** no.(%): right** | 12/18(66.7%) | 10/18(55.6%) | 12/21(57.1%) | 0.76 |
| **Part of foot affected –** no.(%) |  |  |  |  |
| **Anterior sole** | 12(34.3%) | 9(42.9%) | 16(76.2%) | 0.032 |
| **Instep** | 14(40%) | 8(38.1%) | 2(9.5%) |  |
| **Heel** | 5(14.3%) | 3(14.3%) | 3(14.3%) |  |
| **Dorsum** | 0 | 1(4.8%) | 0 |  |
| **Can’t remember** | 4(11.4%) | 0 | 0 |  |
| **Activity at time of puncture –** no.(%) |  |  |  |  |
| **Nothing special** | 13(37.1%) | 15(71.4%) | 10(47.6%) | 0.086 |
| **Housework/Gardening** | 14(40%) | 2(9.5%) | 5(23.8%) |  |
| **Job** | 8(22.9%) | 4(19.1%) | 6(28.6%) |  |
| **Footwear at time of puncture –** no.(%) |  |  |  |  |
| **Slippers only** | 26(74.3%) | 16(76.2%) | 17(81%) | 0.598 |
| **Shoes only** | 4(11.4%) | 1(4.8%) | 2(9.5%) |  |
| **Barefoot** | 4(11.4%) | 1(4.8%) | 1(4.8%) |  |
| **Shoes and socks** | 1(2.9%) | 3(14.3%) | 1(4.8%) |  |
| **Felt puncture? –** no.(%): no | 2(5.7%) | 5(23.8%) | 10(47.6%) | 0.001 |
| **Home remedy?** – no.(%): yes | 33(94.3%) | 13(61.9%) | 13(61.9%) | 0.004 |
| **Type home remedy –** no.(%) |  |  |  |  |
| **Disinfectant** | 12/33(36.4%) | 7/13(53.9%) | 3/13(23.1%) | 0.6 |
| **Black dressing***** | 8/33(24.2%) | 2/13(15.4%) | 4/13(30.8%) |  |
| **Other****** | 13/33(39.4%) | 4/13(30.8%) | 6/13(46.2%) |  |
| **Unprescribed oral antibiotic?** – no.(%): yes | 0 | 1(4.8%) | 0 | 0.236 |
| **Compliant with diabetes Rx at time of puncture –** no.(%): yes | 34(97.1%) | 21(100%) | 18(85.7%) | 0.08 |
| **Smoking status at time of puncture –** no.(%): current | 0 | 0 | 1(4.8%) | 0.259 |
| **Time from puncture to doctor’s visit -** days (%) |  |  |  |  |
| **Less than 2** |  | 16(76.2%) | 9(42.9%) | 0.078 |
| **2 to 5** |  | 4(19%) | 8(38.1%) |  |
| **More than 5** |  | 1(4.8%) | 4(19%) |  |

*Other includes a needle (1), thumbtack (3), tip of a machete (3), stone fragment (3), wood fragment (2) and barbed wire (1).

**For 20 episodes, participants could not remember which foot was affected. None of those episodes required surgical treatment.

***Black dressing is a tar based ointment

****Other includes antibiotic cream (2), antibiotic powder (4), black shoe polish and kerosene.
